# Impact of different orthodontic treatment modalities on Airway: A literature review

**DOI:** 10.12669/pjms.321.8743

**Published:** 2016

**Authors:** Nasser D. Al Qahtani

**Affiliations:** 1 Dr. Nasser D. Al Qahtani, Assistant Professor, Department of Orthodontics and Dentofacial Orthopedics, College of Dentistry, King Saud University, Riyadh-11433, Kingdom of Saudi Arabia

**Keywords:** Airway dysfunction, Orthopedic treatment, Skeletal patterns, Surgical treatment

## Abstract

This review focused on airway dysfunctions and orthodontic treatment modalities. A systematic search of the dental literature was performed using PubMed and Web of Science library database. Different combinations of search terms related to airway and orthodontic treatment were used. Any Non-English articles were excluded. Among titles found, abstract and full articles were reviewed. References from all the relevant articles were hand-searched to include more articles. Forty articles which were found relevant were included in the review. Surgical, orthopedic and fixed appliance therapy has been advocated by clinicians to treat patients with airway dysfunctions. These treatment modalities differ from patient to patient and have to be considered based on lot of criterion.

The reviewed studies were not convincing in providing information about the orthodontic treatment modalities; further research regarding the same could be encouraging.

## INTRODUCTION

The mutual interaction between the pharyngeal structures and the skeletal relationship is a subject of interest for the orthodontists and maxillofacial surgeon. Orthodontists believe that evaluation of soft tissues including facial contours, neuromuscular function, tongue, tonsil, adenoids and nasal polyps should be an integral part of orthodontic diagnosis and treatment planning.^1^ The pharyngeal airway is an intricate structure. In conjunction with its surrounding structures, it is responsible for the physiologic processes of swallowing, vocalization, and respiration.^2^

There was no final conclusion attained in the attempt to establish cause - and - effect relationship between nasal obstruction, craniofacial morphology and occlusal features. Mouth breathing influence the facial form, and is considered a predisposing factor to the development of the “long face syndrome” or “adenoid facies”.^3^ The characteristic of mouth breathing subject includes increased lower anterior facial height, retrognathic mandible, proclined maxillary incisors, high v-shaped palatal vault, maxillary constriction, flaccid and short upper lip, flaccid perioral musculature and dull appearance due to a constant open-mouth posture.^4^ Mouth breathing was correlated with lowered position of the hyoid bone and anterior - inferior postured tongue with significant downward inclination of the mandible.^5^ On the other hand, the typical feature of the long face syndrome was the expression of a hereditary pattern (somatotype) and that mouth breathing was unrelated and should not be considered as an etiological factor and that could only be a para-phenomenon.^6^

Obstructive sleep apnea (OSA) syndrome is characterized by temporary occlusion of the upper airway several times during the night which may result in hypoxia and sleep fragmentation and that the main symptoms were chronic tiredness, day-time somnolence associated with snoring and intellectual deterioration.^7^ A decrease in the upper airway dimension at the velopharyngeal level together with an increase in soft palate and tongue dimensions was observed.^8^ In one of the study it was found that the body mass index of patients was not related to positional changes of the hyoid bone.^9^ The airway impairment has also been associated with syndromes such as Apert’s or Crouzon’s.^10^ Both syndromes were characterized by severe maxillary hypoplasia, which has been suggested as the source of airway obstruction in the affected subjects.

Studies on the changes of upper airway dimensions have consisted of analyzing the post-treatment effects of RME with dental casts, human skull models, 2-dimensional cephalometricradiographs, 3-dimensional imaging techniques including magnetic resonance images, Computed Tomography(CT), Cone-beam computed tomography (CBCT) systems, acoustic rhinometry and computed rhinomanometry.^11^-^13^

Among all the existing 3D imaging techniques, CBCT has become an alternative technique to CT scanning for a comprehensive head and neck evaluation due to its significantly lower overall effective radiation dose and greater spatial resolution than medical CT, high contrast between the hard and soft tissues, lower cost and easier access and availability to dentists.^14^,^15^ Despite the fact that with CBCT, it is not possible to discriminate between the various soft tissue structures, it is possible to determine the boundaries between soft tissues and air spaces making CBCT a potential diagnostic method to analyze airway dimensions.^16^

### Impact of different orthodontic treatment modalities on airway

*a) Surgical Treatment:* Several studies have reported that there was relative narrowing of the airway space after mandibular surgical set-back.^17^,^18^ Other studies have reported that the successful treatment of OSA was found to be associated with the mandibular surgical advancement.^19^,^20^ On the contrary, there were studies which found no difference in the airway size after surgical set back of the mandible.^21^,^22^ It has been reported that there was a lowering in the position of the hyoid bone over time in asymptomatic male controls and after mandibular set-back surgery.^23^ The finding suggested that it could be a physiologic phenomenon related to the increase of airway resistance or airway length. However, the position of the hyoid bone was moved in the opposite direction with mandibular advancement surgery. Lowe et al. mentioned that it is very important to observe the ratio between soft palate and pharyngeal space to preserve correct speech and prevent sleep apnea in later life.^8^ They focused on the treatment of skeletal Class III subjects involving maxillary protraction which was stated to disturb the afore-mentioned balance causing speech difficulty after therapy. In one of the study to evaluate the effect the maxillary advancement or impaction combined with maxillary setback on pharyngeal air way and maxillary sinus volume, it was reported that there was a significant decrease only for lower and total pharyngeal airway volumes in males and a significant decrease in the volume of the maxillary sinuses.^24^

It was found that maxillomandibular advancement increased airway dimensions by increasing the distance from the occipital base to the pogonion. An increase of this distance showed a significant correlation with an improvement in the apnea-hypopnea index and a decreased pressure effort of the upper airway.^25^ Decreasing the pressure effort will decrease the breathing workload. This improves the condition of obstructive sleep apnea syndrome. The upper airway became wider in patients with Class II malocclusion deformity who had undergone mandibular advancement. However, this might become narrower with time.^26^

*b) Orthopedic Treatment:* The sagittal dimension of the upper airway was significantly increased following functional appliance therapy in growing Class II patients.^27^ An increase in the superior upper-airway dimension by maxillary protraction was also observed.^28^ A possible explanation given was that, the maxillary forward growth could bring the tongue to a more anterior position causing the soft palate to come to a more anterior position; thus increasing the upper pharyngeal dimension. In a long term study it was found that the increase in the nasopharyngeal airway of patients with maxillary protraction continued to be significant even after a four-year follow up period while a significant increase in the oropharyngeal space was observed only after the follow-up period.^29^ A mandibular advancement device in sleep apnea patients increased the pharyngeal airway and a reduction of the distance of the hyoid bone to the mandibular plane.^30^ The mechanism of action of the cervical headgear was associated with expansion and an upward and forward rotation of the maxilla.^31^ This could be accompanied by an increase in the upper airway space and could be favorable for breathing. Maxillary expansion was shown to reduce upper airway obstruction during sleep in young adults with mild or moderate obstructive sleep apnea.^32^ Rapid Maxillary Expansion (RME) produced a numerically parallel expansion of the mid palatal suture and a triangular shape of expansion with the base facing anteriorly when percentage change was calculated.^33^ In regard to the airway, a moderate increase of the cross sectional area adjacent to the hard palate was observed. This cross sectional area increase was highly dependent on the expansion between the 1st molars. The RME effect on the airway diminished as it moved further away from the mid palatal suture possibly due to the compensation generated by the surrounding soft tissues in a 3D frame. The treatment with the X-bow appliance in Class II patients resulted in favorable increase in the oropharyngeal airway dimensions and volume.^34^ In one of the study it was found that rapid maxillary expansion and facemask (*RME*/*FM*) therapy did not affect at all the volume of maxillary sinuses and actually inhibited the normal expected increase of the volume of the pharynx when compared with a control group comprising normal individuals.

The RME therapy improved the nasal airway ventilation and was detected by computational fluid dynamics.^35^ Changes noted in the oropharynx may be due to the lack of a standardized position of the head and tongue at the time of image acquisition.

*c) Fixed Appliance Therapy:* There was no study reported in the literature regarding the effect of fixed orthodontic treatment on the airway dimensions. The effect of extraction versus non-extraction treatment on oropharyngeal airway volume with the aid of CBCT showed no significant differences in the oropharyngeal airway volume.^36^ It was found that extraction of the first premolars for the treatment of bimaxillary protrusion did not affect upper airway dimensions despite the significant reduction in tongue length and arch dimensions.^37^ Large incisor retraction leads to narrowing of the upper airway in adult bimaxillary protrusion patients.^38^ The extraction of upper premolars rather than non-extraction in patients decreased the pharyngeal airway space with mandibular prognathism who planned to have bimaxillary surgery.^39^ The Forsus™ Fatigue Resistant Device (FRD) produced dento alveolar changes but did not have any significant influence on posterior airway in young adult patients.^40^

## CONCLUSIONS

The reviewed studies were not convincing in providing information about the impact of different orthodontic treatment modalities on airway; further studies are mandatory regarding the same.
